# Biomarkers in Anderson–Fabry Disease

**DOI:** 10.3390/ijms21218080

**Published:** 2020-10-29

**Authors:** Irene Simonetta, Antonino Tuttolomondo, Mario Daidone, Antonio Pinto

**Affiliations:** Department of Health Promotion, Maternal and Infant Care, Internal Medicine and Medical Specialties, “G. D’Alessandro”, University of Palermo, Piazza delle Cliniche n.2, 90127 Palermo, Italy; mariodaidone@gmail.com (M.D.); antonio.pinto@unipa.it (A.P.)

**Keywords:** fabry, biomarkers, lyso-Gb3

## Abstract

Fabry disease is a rare lysosomal storage disorder caused by a deficiency of α-galactosidase A, resulting in multisystemic involvement. Lyso-Gb3 (globotriaosylsphingosine), the deacylated form of Gb3, is currently measured in plasma as a biomarker of classic Fabry disease. Intensive research of biomarkers has been conducted over the years, in order to detect novel markers that may potentially be used in clinical practice as a screening tool, in the context of the diagnostic process and as an indicator of response to treatment. An interesting field of application of such biomarkers is the management of female heterozygotes who present difficulty in predictable clinical progression. This review aims to summarise the current evidence and knowledge about general and specific markers that are actually measured in subjects with confirmed or suspected Fabry disease; moreover, we report potential novel markers such as microRNAs. Recent proteomic or metabolomic studies are in progress bringing out plasma proteome profiles in Fabry patients: this assessment may be useful to characterize molecular pathology of the disease, to improve diagnostic process, and to monitor response to treatment. The management of Fabry disease may be improved by the identification of biomarkers that reflect clinical course, severity, and the progression of the disease.

## 1. Introduction

Fabry disease is a rare X-linked lysosomal storage disease arising from a deficiency in alpha-galactosidase A (GLA) that results in the accumulation of glycosphingolipids. Anderson–Fabry disease is characterized by a pleiotropic phenotype, including cardiomyopathy, renal failure, vasculopathy, sweating abnormalities, acroparesthesias, angiokeratomas, and gastrointestinal symptoms. Generally, two phenotypes have been recognized: an early-onset classical form and a late-onset form that it is often characterized by a single organ involvement.

In males, diagnosis is provided by the demonstration of a reduced enzymatic αGal activity with supporting genotyping; in females, because of random X inactivation, enzyme activity can result in the normal levels, and genetic analysis of the GLA gene is required to confirm the diagnosis [1].

Recently, consensus initiatives have tried to establish some diagnostic criteria when the pathogenetic effect of a new mutation has not previously been reported. Critical diagnostic points are the demonstration of reduced α galactosidase A activity, significant clinical manifestations, and the detection of substrate accumulation on biopsy [2].

It is known that there is not a specific, definite relationship between an individual GLA mutation and clinical manifestations, and other factors seem to have an impact on disease progression. Additionally, the relationship between genotype and quantitative difference levels of substrates in tissues is still not well understood, thus causing different effects on organ changes [3].

The accumulated Gb3 is converted to lyso-Gb3 by acid ceramidase within tissues, and it is measurable in biological fluids and detectable on histological samples of affected organs [4].

Lyso-Gb3 is also found in patients with late-onset variants associated with instead preserved enzyme activity despite relevant single organ changes [5].

In the context of lysosomal disease, a growing number of sensitive and specific biomarkers is used for screening, for supporting diagnosis, and for monitoring response to treatment.

A biomarker is a measurable analyte that indicates some biological states or processes that are responsible for clinical manifestations of a specific disease. Biomarkers can be measured in different biological samples such as urine, blood, and cerebrospinal fluid; their measurement may be performed before or at the same time as the dosage of enzyme activity.

**Figure 1:**
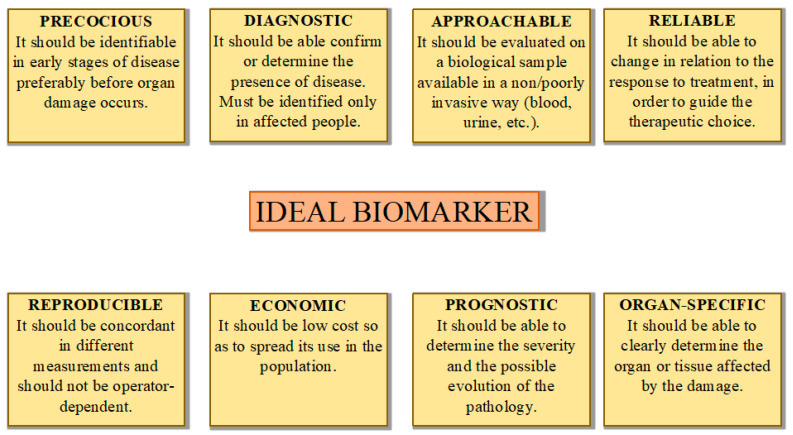
Ideal characteristics of a biomarker in the diagnosis and monitoring of a disease.

Besides having a diagnostic role, biomarkers are useful in order to evaluate the response to treatment, to choose the therapeutic strategy, and to study disease evolution over time. (Figure 1) The analysis of biomarkers may be qualitative or quantitative: qualitative analysis can be carried out through electrophoresis, spot tests, and thin layer chromatography, which are associated with low sensitivity and specificity. Quantitative studies can be performed using the promising tandem mass spectrometric methods and several colorimetric techniques [6].

Several attempts have been carried out over the years in order to identify disease-specific biomarkers that could be potentially used as screening tools as well as indicators of response to treatment.

The analysis of lyso-Gb3 (globotriaosylsphingosine) is preferred to Gb3 (globotriaosylceramide) with regard to Fabry disease. Indeed, several studies have reported and demonstrated the role of lyso-Gb3 as a helpful biomarker for the diagnosis and therapeutic monitoring in patients with Fabry disease; thus, it has not been proven a clear correlation between Gb3 concentrations and the clinical manifestations of the disease [7].

Dried blood spots (DBS) is a valid, sensitive, and reproducible method for measuring lyso-Gb3 levels.

Lyso-Gb3 is the Gb3 degradation product; it is currently measured in the context of disease screening and diagnosis. In fact, it helps in determining the pathogenicity of a mutation [8].

Moreover, it is used to monitor treated patients: it decreases with ERT, as shown in some studies [9,10].

In order to evaluate the organ involvement, numerous practical common biomarkers can be measured as creatinine and proteinuria regarding renal damage, troponin I, and high sensitivity cardiac troponin assays regarding cardiac lesions. Moreover, in the framework of the assessment of cardiac involvement, new cardiac imaging techniques are proving to give valid parameters to detect incipient alterations [11].

The availability of enzyme replacement therapy has demonstrated to have effects on clinical manifestations, laboratory findings, and imaging variables and mortality. Risk analyses suggest that a delay in starting treatment, at older ages or later stages of the disease, is associated with a lower response to treatment, hence the importance to detect disease at a pre-fibrotic stage when it is still possible to optimize outcomes [3].

Peripheral blood mononuclear cells (PBMCs) are an attractive valid alternative to measure Gb3 deposit, thus avoiding invasive methods, and it is also a low-cost alternative method [8].

A study reported a reduction in Gb3 load in PBMCs in patients treated with long-term enzyme replacement therapy. It was described as an efficient method for disease monitoring; however, this measurement on PBMCs was not reported as an excellent alternative to determine Gb3 deposits in patients with non-classical or missense mutations; in particular, the authors reported this finding in subjects with D313Y mutation. [12]

To date, there are limited biomarkers of Fabry disease. The knowledge of the pathogenesis of tissue and organ dysfunction allows us to establish a relationship between a biomarker and the progression of the disease.

## 2. Lyso-Gb3: A Biomarker of Systemic Involvement

Plasma LysoGb3 constitutes an ideal biomarker for the diagnosis of classic Fabry disease. Recent novel use of plasma lyso-Gb3 incorporates the levels of six analogues; however, the accuracy of this measurement did not increase diagnosis in the cohort of patients in the study by Haran Yogasundaram [13].

**Figure 2:**
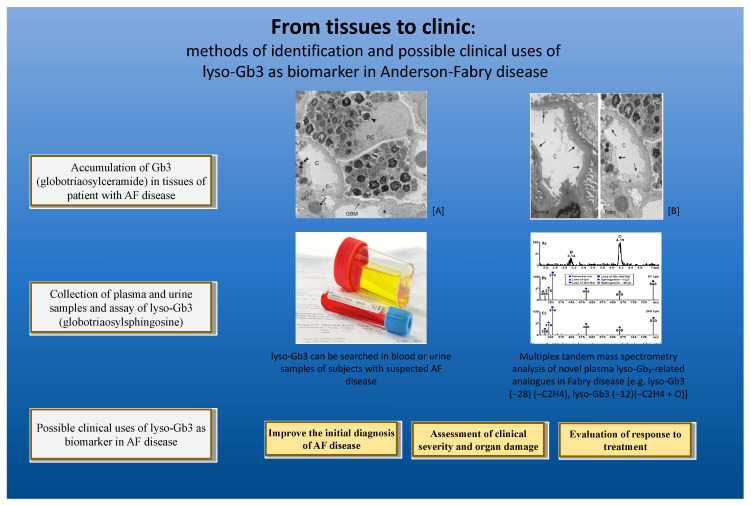
The deacylated form of Gb3, globotriaosylsphingosine (lyso-Gb3), was recently identified as a strong biomarker of Fabry disease. Lysogb3 accumulation can be identified in organs and tissues and can be easily quantified in plasma and urine samples of patients with suspected or documented Fabry disease.(**A**) Globotriaosylceramide (Gb-3) inclusions from the kidney biopsy of a Fabry patient [18]; (**B**) comparison of capillary endothelial coverage in kidney biopsies from a normal subject and a Fabry patient [18].

Several studies evidenced the presence of lyso-Gb3 analogues in urine and plasma (Figure 2). Their structure and empirical formula were determined by mass spectrometry. A correlation between biomarker profiles with clinical manifestations was reported in a group of patients with classical form, late-onset cardiac variant, and atypical mutations [14,15,16,17].

Lyso-Gb3 has been studied on several levels of applicability. In a recent study by Hiroki Maruyama [19], plasma lyso-Gb3 was analyzed as a primary screening marker for classic and late-onset Fabry male and female patients. The authors reported that the effectiveness of plasma lyso-Gb3 screening for selecting subjects that may be submitted to genetic counselling and thus detecting Fabry cases that were not still recognized; moreover, this analysis allowed a reduction in the amount of genetic analysis.

In this study, lyso-Gb3 levels were measured by ultra-performance liquid chromatography/tandem mass spectrometry.

The results suggest the usefulness and applicability of plasma lyso-Gb3 to identify candidates to genetic studies; this approach finally results in the improvement of the outcomes of diagnosis. This analysis also showed difficulty to define a cut-off value in order to classify heterozygous patients. Moreover, this research, as already reported by Rombach et al., supports the importance of lyso-Gb3 as a screening tool for females with left ventricular hypertrophy [20]. Another finding was the opportunity to reveal an association, in female patients, between high lyso-Gb3 levels and normal alpha-Gal A activity with intronic GVUS (genetic variants of uncertain significance), through lyso-Gb3 screening. The detection of high levels of plasma lyso-Gb3 favours the continuation of further analysis, as histological ones (Figure 2), to achieve a definitive diagnosis, thus permitting the selection of patients at high risk of Fabry disease.

Nowak et al. showed an increase in the levels of lyso-Gb3 in females with normal enzymatic activity and who manifested significant clinical characteristic of Fabry disease [7]. Another study described a correlation of lyso-Gb3 levels to imaging signs, in addition to phenotype and genotype [21].

Lyso-Gb3 represents the only available specific biomarker of Fabry disease nowadays. Although it represents an extremely useful tool in the diagnostic process and monitoring of disease, further studies are needed to identify a highly specific biomarker of Fabry disease pathology and treatment outcome (Figure 1).

## 3. Biomarkers of Organ Involvement

### 3.1. Biomarker in Renal Disease

Urine is an excellent biological fluid that can be obtained non-invasively in large amounts.

It is known that Gb3 is elevated in the urine of patients with Fabry disease. Its measurement in urine sediment of a 24-h collection is not advisable and adapted for screening of a large number of patients [22,23,24].

Patients with null mutations or with no residual enzyme activity present elevated levels of urinary Gb3; lower levels are reported in female heterozygotes and those patients with a major residual α-Gal A activity; however, normal levels of urinary Gb3 have also been measured in these last groups of patients [25,26].

A correlation was found between urinary Gb3 and genotype, sex and treatment [27].

Gb3 is a traditional marker that has been measured in urine in order to evaluate the response and efficacy of a specific treatment: it has been found to be reduced in response to ERT; however, it is not a valid surrogate marker in treatment trials.

Urinary Gb3 is released from kidney tubular cells and the urinary collecting system; it has been detected in lysosomes of renal tubular cells; podocytes from Fabry patients are characterized by a dense accumulation of Gb3 [28].

Although levels of Gb3 in urine were used as a biomarker, their clinical and research application were controversial [26]. Its diagnostic role for some patients and screening was reported.

It is an indicator of metabolic effects of ERT: its concentrations reduced in urine two weeks after starting ERT. The production of alpha-galactosidase A antibodies was associated with an increase in urinary Gb3 [29,30].

No evidence has been reported about the prognostic role of urinary Gb3 or its value as a marker for monitoring effects of treatment on nephrological outcome. A study by Auray-Blais [27] showed that estimated GFR was not correlated with urinary excretion of Gb3. Other traditional urinary biomarkers, such as proteinuria and albuminuria, are used in patients with Fabry disease as surrogate markers of glomerular or tubular alterations [31]; they are readily available and outstanding as a predictor of the risk of Fabry nephropathy and its progression. Uromodulin, N-acetyl-β-D-glucosaminidase, and beta2 microglobulin are some other biomarkers of renal dysfunction under investigation [32].

Auray-Blais et al. analyzed urinary metabolites in 63 untreated Fabry patients and 59 healthy subjects. Seven novel urinary biomarkers (all lyso-Gb3 analogues having modified sphingosine moieties) were detected in Fabry patients by performing multivariate statistical analysis; none of these markers was revealed in controls [14]. Tandem mass spectrometry analysis was performed on concentrated urine samples in order to obtain information about the structure of these novel seven potential Fabry biomarkers; notably, they showed fragmentation profiles similar to lyso-Gb3. The identification of lyso-Gb3 and its analogues can be an essential diagnostic aid as they have been identified only in cases and not in controls.

Further analysis of the different plasmatic lyso-Gb3 analogues may provide additional explanations on the tissue of origin of the Gb3 from which they are derived.

The levels of these new biomarkers were higher in male patients compared to female patients; these findings suggest that the urinary extraction of these specific markers correlates with clinical severity. In fact, in females, the phenotype is less severe than in affected men. This metabolomic study revealed that all lyso-Gb3-related analogues showed a reduced urinary extraction in male Fabry patients after treatment compared to untreated patients, thus suggesting the applicability of these compounds for the pre-treatment follow up and monitoring the response to therapy. A larger sample of patients is required in order to evaluate the correlation between biomarkers’ disease severity and progression. Future innovative methodologies will quantify all these analogues on a triple or quadrupole mass spectrometer [14,27,33].

### 3.2. Biomarkers in Vascular and Cardiac Involvement

The vasculopathy is the underlying cause of the main severe complications of the disease that are hypertrophic cardiomyopathy, stroke, and renal failure. Several animal models of vascular disease have been used as the Gla-null mouse exhibiting several inducible vascular phenotypes as accelerated atherogenesis, impaired vasorelaxation, and oxidant-induced thrombosis. The endothelial nitric oxide synthase (eNOS) dysfunction is a common mechanism which potentially links these three vascular abnormalities. The alteration of this enzyme (eNOS) causes reduced nitric oxide bioavailability, enzyme uncoupling resulting in the generation of peroxynitrite, and a reactive nitrogen species [34,35,36,37].

Liming Shu et al. generated a human cell model in which hybrid endothelial cells were transfected with siRNA (small interfering RNA) directed against alpha-galactosidase A. In these cells, the enzyme activity of eNOS was reduced by > 60%. This study aimed to evaluate whether induced changes in Gb3 cellular content would be enough to consider eNOS abnormalities.

Previously, other authors pointed out that 3-nitrotyrosine (3-NT) may be considered as a marker of cardiovascular disease in humans [38,39].

Under the established experimental conditions, the authors showed an uncoupling of eNOS with the production of 3-nitrotyrosine in the presence of increased Gb3 content in EA hy926 with GLA knockdown. 3-nitrotyrosine is a specific marker for reactive nitrogen species. They measured it through mass spectrometry, and they found that it was increased by 40- to 120-fold. This figure was not associated with corresponding changes in other oxidized amino acids. Moreover, they observed high 3-NT levels in the plasma and aortic extracts in the Gla knock-out mice. These findings confirmed the eNOS uncoupling.

Furthermore, they were not observed with beta glucocerebrosidase knockdown. Indeed, a reduction in eNOS activity was reported, and it was compatible with the altered production of NO. The researchers completed their investigation by measuring the levels of protein-bound oxidized amino acids in plasma samples from patients with classic phenotype and compared the results with age and gender-matched controls. They found elevated 3-NT levels in the absence of similar changes in other oxidized amino acids [40].

This study suggests the potential use of 3-NT as a biomarker of vascular involvement in Fabry disease. It may also be used in a prognostic sense, to establish the risk of manifesting vascular complication, and finally to monitor the response to current treatment to reduce Gb 3 accumulation. 3NT levels were significantly high in knock-out mice, above all elevated in older ones. On the other hand, a relevant increase in other tyrosine adducts (meta-tyrosine, di-tyrosine, etc.) was not observed in plasma or aortic samples from the Gla null mice [41].

In light of the above, eNOS uncoupling is detected in the presence of GLA deficiency, and it is a potential expression of altered vascular function [40].

Further research is needed to confirm these results through prospective clinical studies and by extending studies to a larger number of patients, including non-classical phenotypes, too.

Cardiovascular manifestations of Fabry disease include LVH (left ventricular hypertrophy), diastolic dysfunction, valvulopathy, microvascular angina, and conduction defects [42,43].

In classical phenotype, cardiac symptoms and signs typically occur between 30 and 40 years resulting in heart failure [44,45].

Several proteins and cytokines have been studied as factors participating in cardiac disease, and some of them are studied as plasma markers that may have value in prognosis and knowledge of the pathophysiology of the disease [46].

Haran Yogasundaram et al. [13] compared plasma concentrations of several biomarkers in adult patients with Fabry disease to those of healthy controls to obtain a significant awareness of the burden of disease of cardiac involvement and their link to heart failure with preserved ejection fraction (HFpEF). In this study, inflammatory and cardiac remodelling markers were measured from the plasma of 68 Fabry patients and 40 controls. TNF (tumour necrosis factor), IL-6 (interleukin-6), TNFR1 (tumour necrosis factor receptor 1), and TNFR2 were significantly elevated in Fabry subjects indicating the leading role of chronic inflammation in the pathophysiology of the disease. It is known that TNF and IL-6 are elevated in heart failure, and its levels present a correlation to reduced functional condition and all-cause mortality [47,48].

This study pointed out a positive correlation between inflammatory biomarkers with disease severity evaluated through the Mainz severity score index (MSSI), cardiac-specific scores, left ventricular hypertrophy, diastolic dysfunction, and renal disease supporting the critical role of inflammation and vascular dysfunction in the pathogenesis of HFpEF [49,50].

Moreover, this study reported a significant association between elevated plasma concentration of inflammatory markers with renal damage in the studied cohort. These are relevant data in consideration of the known link between renal disease and HFpEF [51].

Both TNFR1 and TNFR2 were found to be increased, and they were associated with late gadolinium enhancement (LGE) in cardiac MRI, which is an early sign of cardiac hypertrophy; thus, they may be used to detect pre-hypertrophic stages allowing the opportunity of selecting those patients to further investigate through cardiac MRI [52,53]. Currently, these receptors are studied as potential targets of novel therapies in the management of heart failure [54,55].

Levels of BNP and MR-proANP are higher in Fabry patients with LGE and diastolic dysfunction: this finding suggests the existence of a relevant long term pathological cardiac remodelling. Another two proteins that are implicated in remodelling are MMP2 (matrix metalloprotease 2) and MMP9; in particular, it was reported that elevated levels of MMP2 were associated with HFpEF, and on the other hand, MMP9 levels were found to be increased in LVH and detrimental extracellular matrix remodelling [56].

Moreover, a correlation of MMP2 with MSSI and other signs of cardiomyopathy in Fabry disease were reported (LVH, LGE, diastolic dysfunction).Additionally, elevated levels of galectin-1 supported the significant role of extracellular matrix remodelling in Fabry disease. Data about galectin-3 suggested its role as a biomarker of advanced disease and progression of heart failure [57].

Despite enzyme replacement therapy, dysregulation of inflammatory processes persists in some patients, for which it may be helpful to measure levels of some biomarkers in order to obtain information disease control and prognosis.

The study of proteomic biomarker platforms will help to detect HFpEF phenotypes to provide an appropriate choice of phenotype-specific treatment [58].

Monitoring markers of cardiac remodelling and systemic inflammatory may provide increased sensitivity for detecting early subclinical manifestations and thus for selecting patients that may benefit from an early beginning of ERT to prevent complications [59]

Among potential circulating biomarkers, some substrates and derivatives and downstream markers of pathophysiology and markers of fibrosis have been evaluated: lyso-GB3 and matrix metalloproteinase 9 were higher in Fabry disease and NT-proBNP (N terminal of the prohormone brain natriuretic peptide, imaging markers) [34,60,61].

Cardiac magnetic resonance provides essential data about cardiac involvement in Fabry disease as the presence of posterior wall late gadolinium enhancement corresponding to myocardial fibrosis is a typical postmortem finding within the posterior wall.

A study by Niemann et al. [62] reported the association between late gadolinium enhancement with increased left ventricular mass, worsening in myocardial function and failure to respond to treatment. Another study showed some relevant factors associated with myocardial dysfunction including a right ventricle (RV) free wall thickness greater than 7 mm, left ventricular (LV) longitudinal dysfunction, and left ventricular septal wall thickness greater than 15 mm. Moreover, the analysis of LV and RV strain pointed out that left ventricular and right ventricular fibrosis was associated with reduced LV and RV strain [63]. Non-contrast T-1 mapping is a recent method as part of cardiac magnetic resonance imaging in Fabry disease: it is based on the knowledge that lipids lower the T1 parameter. Thus, it may allow the early detection of cardiac involvement. T1 also gives the possibility of differentiating other causes of left ventricular hypertrophy (LVH) such as hypertension, amyloidosis, and hypertrophic cardiomyopathy. It was reported that low T1 occurred early in some patients before development of evident hypertrophy, so it can be considered a marker of incipient heart damage and an imaging marker that may be useful to evaluate therapeutic effects.

Another image parameter used in the cardiological evaluation of Fabry patients is the regional longitudinal strain (LS), an echo-doppler cardiac parameter. Esposito et al. [64] recently reported an association between higher lyso-Gb3 levels and lower apical longitudinal strain values in Fabry patients. According to the results of this study, the regional LS distribution may be caused by the accumulation of lyso-Gb3 in myocytes.

## 4. Other Biomarkers

Proteins are nowadays considered as a potential biomarker in many diseases. Therefore, the proteomics approach is among the most promising approaches in this field.

The objective of recent studies is that of identifying novel Gb3 isoforms through metabolomic analysis or proteomic studies [65,66].

Previous studies were conducted to find new biomarkers with several efforts to detect metabolites that may assess the effectiveness of ERT to monitor disease progression. A recent study analyzed plasma proteome profiles before and after ERT in order to better define the molecular pathology of Fabry disease [67].

To date, a comprehensive proteomic and metabolomic analysis on Fabry patients has not been performed; only recently, the application of a proteomic approach on peripheral blood mononuclear cells (PBMC) from Fabry patients has allowed the detection of some specific Fabry markers as calnexin, g-enolase, and galectin-1 [68].

Matafora et al. studied the urinary proteome of Fabry naive patients. They found an up-regulation of uromodulin, prostaglandin H2 d-isomerase, and prosaposin in the urine of these patients. Moreover, they demonstrated the correlation between the identified markers and the efficacy of the Fabry specific-therapy. Additionally, the authors identified several pathways, such as those related to glycolysis, lysosome metabolism, and sphingolipid metabolism that were up-regulated in Fabry patients compared to healthy subjects. In particular, they reported elevated expression of beta-galactosidase in Fabry naive patients, suggesting the role of this molecule in the process of accumulation of glycosphingolipids [66].

Sun Hee Heo and colleagues used two-dimensional electrophoresis, MALDI-TOF MS (matrix-assisted laser desorption/ionization-time of flight tandem mass spectrometry), and tandem mass spectrometry (MS/MS) in plasma samples before ERT and then at 3 to 12 months after ERT. They identified proteins in the plasma of Fabry patients with classical phenotype; in particular, they investigated the changing levels of these proteins during a short period of treatment; these values were then compared to samples of a cohort of gender- and age-matched controls. Among the several abundant plasma proteins, most of the proteins such as albumin, antitrypsin, and transferrin were removed through MARC; the authors chose those proteins which were detected by both MS and MS/MS methods, particularly the levels of 15 proteins showed a reduction after ERT and no protein showed increased levels after ERT. These identified proteins included factors involved with inflammation, tissue remodelling, angiogenesis and atherosclerosis, complement pathway, DNA repair, and folding of proteins.

Five proteins (ACTB, the ACTB binding protein, and pFN1) and some components of complement pathways (C1QC, C3, and C4) were selected to analyze their different levels after treatment. As regards in vivo characterization of complement status, the authors analyzed renal histological samples before ERT finding an immunofluorescent positivity of IgM and C3 deposits in seven and 8 patients.

The results of this study underlined that the evaluation of the functional roles of the selected proteins and other changes of their levels throughout enzyme replacement therapy is relevant in order to potentially use these proteins as biomarkers of the effectiveness of ERT. The results showed interactions between ACTB and PFN1-NOS3 or eNOS as well as the complement pathway suggesting their potential role as biomarkers of the molecular pathology of Fabry disease: ACTB is involved in the eNOS regulation [69], and PFN1 participates in actin polymerization as well as VCL. Thus, they may take part in eNOS dysfunction, which is a cause of vasculopathy in Fabry disease [35,70]. A limit of applicability of ACTB as biomarkers was related to the influence of several factors such as fasting, hypoxia, and diet in its levels [71]. As regards C1q, C3, and C4, other regulators of the complement pathway such as CPN1, KRT, and FGA were found to be differentially abundant after ERT: the plasmatic levels of C3 and C4 were augmented in untreated patients, and they showed a gradual reduction during the period of ERT; on the other hand, the levels of C1QC did not vary throughout treatment. Moreover, the alteration of C3 activity was documented by high levels of iC3b in the pre-treatment plasma of a mouse model and by an increase in C3 deposits in pre-treatment human renal histological samples.

C3 deposition was likely induced by renal insult caused by Gb3 accumulation resulting in further renal damage. This study showed that measuring levels of iC3b is helpful to assess the therapeutic efficacy of ERT and also showed a correlation between its change and the cumulative dose of ERT compared to the changes in plasma Gb3. This study indicated the applicability of iC3b as a biomarker of efficacy of ERT and of monitoring the clinical course of the disease; however, plasma iCb3 had some limitations as a diagnostic biomarker above all because its pre-treatment levels were variable in the plasma of Fabry patients. Additionally, it was difficult to establish whether iCb3 changes reflect the immune response to enzyme therapy and whether its reduction suggested the stabilization of the complement activity during ERT. These findings suggest that the complement pathway takes part in the pathogenesis of the disease, and ERT reduces further activation of the complement pathway [67].

Furthermore, this research reported differential levels of ITIH4 (an anti-inflammatory protein), SAA1 (an acute-phase protein), and ENO1 (a tissue remodelling factor indicating the contribution of ischaemic vascular pathology in Fabry disease) [72,73].

Plasma proteomic profiles, although not yet widely used in clinical practice, described before and during treatment, indicate several molecular characteristics of the pathogenesis of the disease. A more detailed extensive analysis of the role of complements and other factors involved in inflammation, oxidative stress, and ischaemic injury and of histological findings is required to determine the contribution of every single pathway and factor in the pathogenesis and its applicability to monitoring disease progression and effectiveness of ERT.

Neutralizing antibodies, which constitute an important issue around ERT, have been recently studied for their impact on clinical outcomes [8]. A Japanese study reported a correlation between antibody levels and lyso-Gb3 levels. Another study showed elevated lyso-Gb3 levels in antibody-positive patients, and this correlation was also associated with worse disease severity as demonstrated using severity scores [10,74,75]. Bènichou et al. carried out a study on samples of skin biopsies from patients receiving ERT. The authors described an impaired Gb3 clearance in cutaneous tissue in those treated patients with higher antibody titres [75].

### MicroRNAs

**Figure 3:**
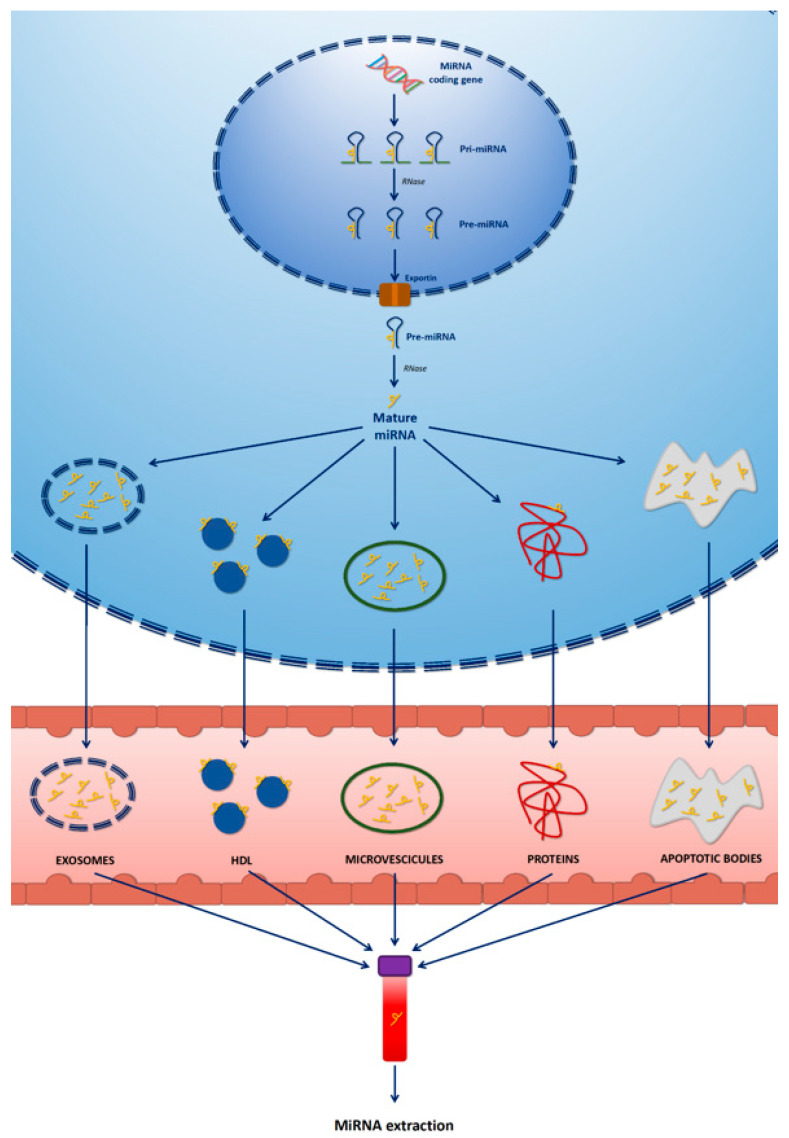
Secretion of miRNAS from nucleus to extracellular spaces and circulatory system. Pre-miRNA and mature miRNAs are secreted from the either in micro particle free form bound with proteins or in encapsulated macrovesicles. There are five main modes by which miRNAs are secreted into extracellular spaces and circulatory system (exosomes, HDL, microvescicules, protein-bound, and apoptotic bodies).

MicroRNAs (miRNA, miR) are small RNA molecules that have been recently investigated in different disease settings, and they have been reported to have a key role in cardiac function [76,77]. They represent potential biomarkers in an early diagnostic phase and to evaluate treatment response. (Figure 3). Although the diagnostic and prognostic utility of miRNAs is widely recognized, the use of these biomarkers is currently only for research purposes.

Cammarata et al. detected four specific microRNAs, of which two were indicators of endothelial dysfunction in Fabry patients. This study was conducted on a limited number of subjects [78].

Recently, Ke Xiao et al. analyzed circulating microRNAs in 6 Fabry patients through sequencing by the HTG EdgeSeq System, and they assessed whether ERT might influence the levels of individual microRNAs. In particular, the authors compared the microRNA pattern of patients receiving ERT with patients who are not in treatment. The pathways that were investigated were axon guidance signalling pathways and the TGF-beta signalling pathway, which were found to be targets of miRNAs. It was reported that abnormal regulation of miRNAs resulted in changes in axon guidance pathways: this effect may contribute to neurological processes leading to neurological manifestations in Fabry disease [79].

The role of circulating miRNAs as biomarkers has been assessed both in kidney disease and Fabry disease. In this regard, Neal et al. reported that plasmatic levels of miR-21 and miR-210 were reduced in patients with chronic renal disease [80]. A recent case report showed reduced expression of miR-29 and miR-200 in urine in a Fabry patient without renal disease: this finding was not reported for other TGF- β related miRNAs. In light of these studies and of other reporting sometimes conflicting results, currently, it is not possible to support the role of TGF β-regulated miRNAs in the field of enzyme replacement therapy [79].

The results of this study by Ke Xiao, through a pathway enrichment analysis, showed the involvement of many miRNAs as miR21-5p and miR19a-3p in the TGF- β signalling pathways. Besides the small size of the studied population, another limitations of this study is selection bias; in fact, this study includes both male and female patients with variable manifestations and the possible different source of circulating mRNAs that may derive from diversified cell type and tissues. Future studies are needed with a larger patient cohort and proper healthy control in order to improve the specificity of the results and to better understand the involvement of miRNA patterns in the underlying mechanisms of effects of treatment.

## 5. Biomarkers and Disease Severity

Auray-Blais et al. investigated the relationship between Fabry biomarkers and disease severity; mainly, they reported a positive association between plasmatic levels of lyso-Gb3 and its urinary analogue levels adjusted for gender and age with LVM index and Mainz Severity Score index [81]. In this regard, Auray-Blais et al. studied lyso-Gb3 and Gb3 profiles in a cohort of patients affected by late-onset cardiac variant. Mass spectrometry was used to measure lyso-Gb3 and seven analogues in urinary samples of these 191 patients. The analogues are characterized by differences in the sphingosine moieties lyso-Gb3 (−28) (–C2H4), lyso-Gb3 (−12)(–C2H4 + O), lyso-Gb3 (−2) (–H2), lyso-Gb3 (+14) (–H2 + O), lyso-Gb3 (+16) (+O), lyso-Gb3 (+34) (+H2O2), and lyso-Gb3 (+50)(+H2O3) [81]. The authors divided patients into four subgroups: adult males, adult females, paediatric males, and paediatric females. They evaluated the sensitivity for Gb3, lysoGb3, and lyso-Gb3 analogues in each subgroup of patients carrying the IVS4 mutation. The authors found a high value of plasma lyso-Gb3 in 15 patients of the adult female subgroup, while 56 patients had at least one abnormal biomarker. The paediatric female group had at least one abnormal biomarker, and only 9% of patients had abnormal levels of lyso-Gb3. In total, 83% of patients of the paediatric male group were found to have at least one abnormal plasma biomarker, while only 14% had an abnormal lyso-Gb3 level. These results underline the importance and utility of analyzing lyso-Gb3 analogues in order to improve the diagnosis in these subjects carrying this genotype associated with a late-onset cardiac variant [81].

The results of this study showed that gender and age are revelated to disease severity and several biomarker levels. The disease was indicated using the total MSSI. It was reported a positive association between several biomarkers and left ventricular mass index and MSSI in adults when adjusting for age and gender. Some of the children with high levels of these biomarkers had a family member that presents a more significant disease severity that other Fabry patients in the same age range. It would be interesting to perform an accurate follow-up of these children to define if the biomarker profile at a young age may be predictive of the clinical outcomes in adulthood [81].

In addition to MSSI and the Fabry Disease Severity Scoring System (DS3) [82,83], recently Mignani et al. [84] developed a novel dynamic model, the FAbry STabilization indEX (FASTEX) to quantify the burden of disease and its clinical stability.

This new system aims to objectively prove whether patients remain clinically stable or whether they become unstable over time.

To generate FASTEX, two scores need to be produced: raw score (RS) and Weighed Score (WS). RS takes into account three domains (nervous system domain, renal domain, and cardiac domain). To check the variation of the disease burden over time, the RS should be repeated after one year. The second score, the WS, is based on the individual items of the RS, and it reflects the degree of organ damage for each field.

According to the results of this work, a FASTEX score ≥ 20% is indicative of clinically significant deterioration, while a score of < 20% suggests stability or improvement of clinical status.

In the context of the assessment of the disease severity and stability, more extensive data from large populations are needed to guide the development of score systems that could be better interpreted in combination with traditional and emerging biomarkers.

## 6. Conclusions

In the context of lysosomal disease, a growing number of sensitive and specific biomarkers are used for screening, for supporting diagnosis, and for monitoring response to treatment. (Table 1) The development of innovative, valid convenient methods for the measurement of biomarkers, such as DBS, will allow more feasible new-born screening for numerous lysosomal disorders. Pedigree analysis of an X-linked disease is informative of genetically at-risk subjects. Genetic screening of younger members of a suspected family allows an early diagnosis, providing also the opportunity to perform a comprehensive, in-depth assessment and to start therapy in those individuals who may come to medical attention after the occurrence of a severe clinical event.

Numerous studies have demonstrated the role of lyso-Gb3 as an accurate biomarker of disease activity, as a screening tool of diagnosis, and as a marker of response to therapy. However, to date, no long-term data are available about clinical outcomes of patients achieving lyso-Gb3 decrease.

As mentioned above, plasma lyso-Gb3 is a promising screening biomarker to select Fabry probands. It potentially allows the recognition of many female unrecognized probands. The dosage of lyso-Gb3 may constitute an excellent approach to confirm diagnosis in females and late-onset patients.

The importance of detecting a marker able to assess disease progression, and to monitor response to treatment, results in the opportunity to evaluate a potential therapy modification. Currently, patient history, careful clinical assessment, and organ investigations are the best tools in clinical practice in addition to targeted therapy for specific organ damage.

The studies reported in this review have highlighted the presence of elevated levels of lyso-Gb3, TNF, TNFR1, TNFR2, and MMP2 in plasma of male patients compared to the levels detected in female patients, which is a consequence of the X-linked inheritance pattern. Increased plasma biomarkers of remodelling such as BNP and galectin-3 were found in older patients that did not present higher levels of inflammatory biomarkers. These findings suggest that ERT may have limited effects in this group of patients; in fact, with an advanced disease in older patients, it is known that it does not give benefits in terms of the prevention of organ failure and death [59].

Markers of inflammation and cardiac remodelling are elevated in those patients with more severe disease assessed with a severity score, such as MSSI, and cardiac-specific scores.

Patients with renal involvement are characterized by elevated plasma levels of biomarkers of extracellular matrix remodelling and cardiac remodelling inflammation.

The plasma levels of inflammatory biomarkers, lyso-Gb3, and cardiac remodelling biomarkers are more elevated in those patients with more severe disease—for example, in the presence of left ventricular hypertrophy and renal failure, this correlation suggests the role of significant states of inflammation in the pathogenesis of multisystem damage from the disease.

A revolution in the management of Fabry disease will be obtained through new advances in the phenotypic specific anti-inflammatory treatment.

The main limitation of all the studies mentioned above is the sample size, principally caused by the rare diagnosis of Fabry disease. The small sample limits the generalization of the results and the quality of statistical tests.

The results of the recent studies regarding the correlation between scores of disease severity and biomarker levels need to be validated in a large cohort of Fabry patients including children, thus allowing the assessment of the usefulness of available biomarkers (Lyso Gb3 and its analogues) as predictive values, in the evaluation of disease severity and the possibility of a relationship with different genotypes.

In light of the new knowledge and of the recent data about the clinical, laboratory, and therapeutic follow up after 15 years of the introduction of ERT, future investigations aim to detect early stages of the disease and specific treatment to apply in a pre-fibrotic state in order to optimize the outcome of Fabry disease.

## Figures and Tables

**Table 1:** Biomarkers and their potential clinical utilities in Anderson-Fabry Disease.

Organ Involvement	Biomarkers	Potential Clinical Utility	Ref.
Systemic	Lyso-Gb3	Diagnosis, disease activity	[7,8,9,10,13,14,15,16,17,18,19,20,21]
	Urinary lyso-Gb3	Diagnosis, disease activity, efficacy of treatment	[22,23,24,25,26,27,28,29,30]
	Proteomic analysis of plasma samples or urinary samples pathways of: □ Inflammation (ITIH4, SAA1) □ Tissue remodelling (EN01) □ Angiogenesis □ Atherosclerosis (eNOS) □ Complement pathway (C1QC, C3, C4) □ DNA repair □ Protein folding	Effectiveness of ERT, monitor disease progression, organ damage	[65,66,67,68]
	Neutralizing antibodies	Prognosis, disease severity	[8,10,73,74]
	MiRNA (miR21, miR210, miR29, miR200, miR21-5p, miR19a-3p, etc.)	Diagnosis, disease activity, response to treatment, prognosis	[75,76,77,78,79]
Kidney	Proreinuria, albuminuria, eGRF	Renal involvement	[31]
	Uromodulin, N-acety-β-D-glucosaminidase, beta2 microglobulin	Renal involvement (under investigation)	[32]
	Urinary lyso-Gb3 analogues	Diagnosis (detectable in case not in control)	[14]
Heart	3-NT	Cardiac and vascular involvement	[38,39,40,41]
	Longitudinal strain distribution	Cardiac involvement, prognosis	[63]
	TNF, IL-6, TNFR1, TNFR2	Cardiac involvement, prognosis	[47,48]
	Cardiac-specific scores, left ventricular hypertrophy, diastolic dysfunction	Cardiac involvement, prognosis	[42,43,49,50]
	Late gadolinium enhancement on cardiac MRI, Non-contrast T-1 mapping	Cardiac involvement (detect pre-hypertrophic stages), prognosis	[52,53,54,55,62,63]
	NT-proBNP, BNP, MRproANP, MMP2, MMP9, galectin-1, galectin-3	Cardiac involvement (remodelling. Diastolic dysfunction), prognosis	[56,57,58,59,60,61]

Lyso-Gb3: lyso-globptriaosyceramide; MiRNA: microRNA; eGRF: Estimated glomerular filtration rate; ERT: enzyme replacement therapy; ITIH4: Inter-Alpha-Trypsin Inhibitor Heavy Chain 4; SAA1: Serum Amyloid A1; ENO1: Enolase 1; eNOS: endothelial nitric oxide synthase; 3-NT: 2-nitrotyrosine; TNF: tumor necrosis factor; IL: interleukin; TNFR: tumor necrosis factor receptor; MRI: magnetic resonance imaging; NT-proBNP: N-terminal pro-B-type natriuretic peptide; BNP: B-type natriuretic peptide; MRproANP: mid-regional pro-atrial natriuretic peptide; MMP: matrix metalloproteinases.
